# A SCOPING REVIEW OF MACHINE LEARNING STUDIES ON DEMENTIA RISK FOR ETHNORACIAL MINORITIES

**DOI:** 10.1093/geroni/igae098.3620

**Published:** 2024-12-31

**Authors:** Michin Hong, Soo-Yeon Ji, Seon Kim, Kyeongmo Kim

**Affiliations:** Indiana University Indianapolis, Indianapolis, Indiana, United States; Bowie State University, Bowie, Maryland, United States; Drexel University, Philadelphia, Pennsylvania, United States; Virginia Commonwealth University, Richmond, Virginia, United States

## Abstract

This study aims to provide an integrated review about dementia risk for ethnoracial minorities from machine learning (ML) based-studies. While ML methods are widely used in dementia research, their benefit for ethnoracial minorities remains unclear. We located prior research using combinations of the key words related to ML (e.g., neural network, deep learning, artificial intelligence), dementia and ethnoracial minorities from major electronic databases. Out of 599 initially searched articles, 389 remained after removing duplicates, and finally, 14 met the inclusion criteria. The interdisciplinary research team employed reiterative data analysis to identify key themes. All included studies, published since 2020, aimed to identify essential risk factors and generate predictive models for cognitive impairments. Most studies and treated race as a predictor after aggregating it into two or three categories, finding a lower risk of dementia among non-Hispanic Whites compared to Hispanics or Non-Hispanic Blacks. One study aimed to develop a predictive model of cognitive impairment applicable across non-Hispanic White, no-Hispanic Black, and Hispanic groups. Various ML techniques were used, such as neural network, random forest, support vector machine, and gradient boosting with some adopting Synthetic Minority Over-sampling Technique to oversample minority groups. Our review indicated that ML-based studies have made limited progress in understanding dementia risk among ethnoracial minorities. National data sets used in most reviewed studies, have lacked minority samples, and this seems to persist with the ML approaches, possibly contributing to deteriorating the existing racial gap. Proactive efforts should focus on ethnoracial variations in dementia risk in ML-based studies.

